# Supplementary material for: High-Pressure Structural Evolution of Na2ZrSi2O7 and Na2ZrSi2O7·H2O: Topology-Driven Compression Behaviors, Phase Stability, and Electronic Transitions
Source: Inorg Chem. 2025 Dec 8;64(50):24594–600. doi: 10.1021/acs.inorgchem.5c04119 (PMC12728915; doi:10.1021/acs.inorgchem.5c04119)
Supplement: Supplementary file 1 [file ic5c04119_si_001.pdf]

Supporting Information

High-Pressure Structural Evolution of $Na_2ZrSi_2O_7$ and $Na_2ZrSi_2O_7 \cdot H_2O$: Topology-Driven Compression Behaviors, Phase Stability, and Electronic Transitions

Peijie Zhang,[1,2,*] *Pablo Botella,*[1] *Neha Bura,*[1] *Xiao Dong,*[3] *Catalin Popescu,*[4] *Yellampalli Raghavendra,*[5] *Rakesh Shukla,*[6] *Srungarpu Nagabhusan Achary,*[6] *Daniel Errandonea*[1]

[1] Departamento de Física Aplicada-ICMUV-MALTA Consolider Team, Universitat de Valencia, 46100 Valencia, Spain;

[2] Center for High Pressure Science and Technology Advanced Research (HPSTAR), 100193 Beijing, China;

[3] Key Laboratory of Weak-Light Nonlinear Photonics, School of Physics, Nankai University, 300071 Tianjin, China;

[4] CELLS-ALBA Synchrotron Light Facility, Cerdanyola, 08290 Barcelona, Spain

[5] Water and Steam Chemistry Division, Bhabha Atomic Research Center (BARC)-Facility, 603102 Kalpakkam, India

[6] Chemistry Division, Bhabha Atomic Research Center (BARC), 400085 Mumbai, India

E-mail: peijie.zhang@uv.es (P. Z.)

Section S1. Supplementary Figures

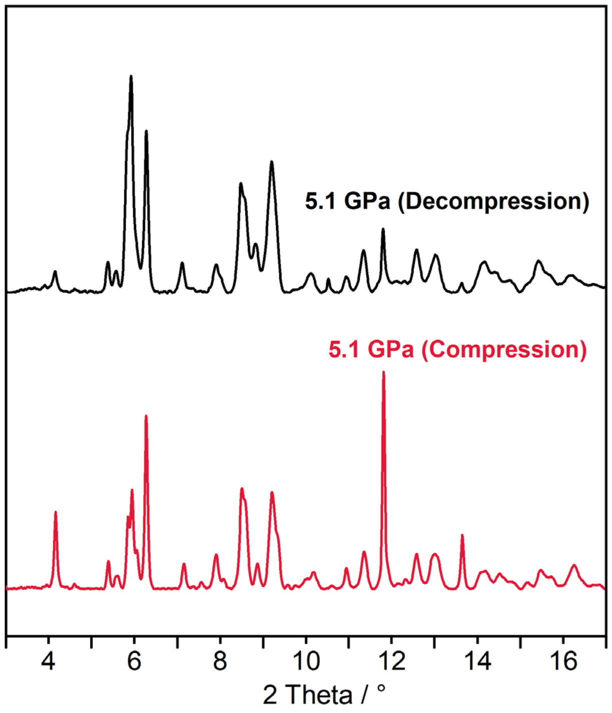

Figure S1. Comparison of in situ HP XRD patterns of $Na_2ZrSi_2O_7$ at 5.1 GPa during compression and decompression.

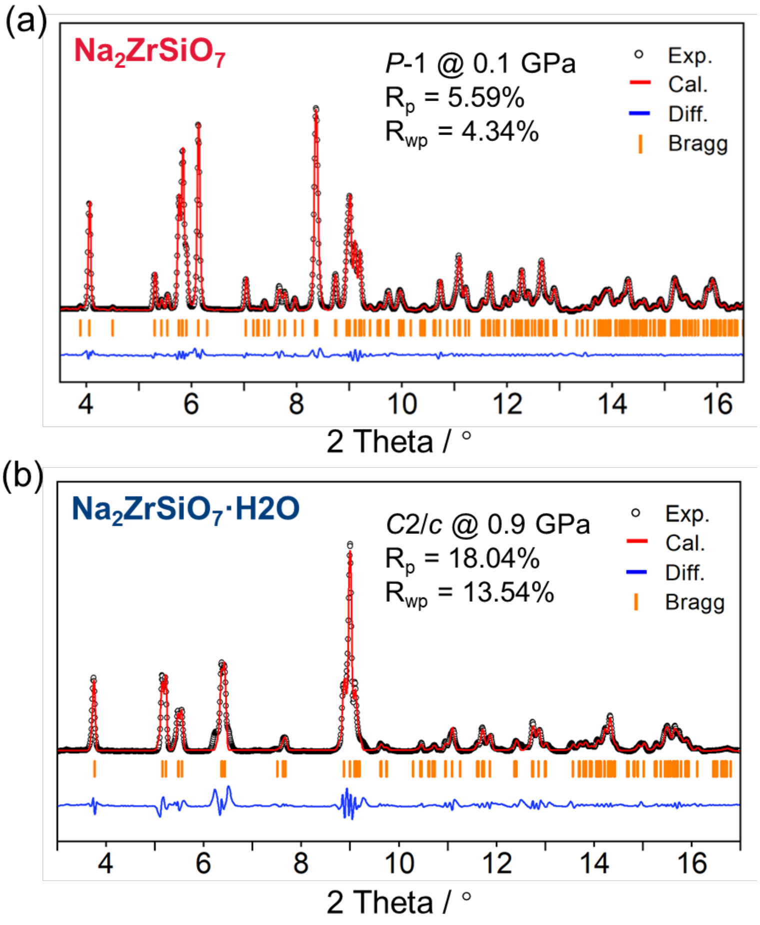

Figure S2. Selected Le Bail fit plots of (a) $Na_2ZrSi_2O_7$ and (b) $Na_2ZrSi_2O_7 \cdot H_2O$ at 0.1 GPa and 0.9 GPa, respectively. The black circles, solid red line, solid blue line, and orange bars represent the experimental data, simulated data, difference, and Bragg positions, respectively.

Section S2. Supplementary Data Analysis

The crystal densities were calculated using the structural information reported in the CIF files. The density (ρ) was determined using the formula:

$$\rho = \frac{M \times Z}{N_A \times V}$$

where M is the molar mass of the formula unit, Z is the number of formula units per unit cell, N_A is Avogadro's number (6.022×10^{23}), and V is the unit-cell volume.

Table S1 Crystallographic parameters for density calculations.

Compounds	$Na_2ZrSi_2O_7$	$Na_2ZrSi_2O_7 \cdot H_2O$
M (g/mol)	305.38	323.38
Z	2	4
V (Å^3)	302.756	673.862
ρ (g/cm^3)	3.35	3.17
